# *Tribolium* beetles as a model system in evolution and ecology

**DOI:** 10.1038/s41437-021-00420-1

**Published:** 2021-03-25

**Authors:** Michael D. Pointer, Matthew J. G. Gage, Lewis G. Spurgin

**Affiliations:** School of Biological Sciences, University of East Anglia, Norwich, UK

**Keywords:** Model invertebrates, Evolution, Ecology

## Abstract

Flour beetles of the genus *Tribolium* have been utilised as informative study systems for over a century and contributed to major advances across many fields. This review serves to highlight the significant historical contribution that *Tribolium* study systems have made to the fields of ecology and evolution, and to promote their use as contemporary research models. We review the broad range of studies employing *Tribolium* to make significant advances in ecology and evolution. We show that research using *Tribolium* beetles has contributed a substantial amount to evolutionary and ecological understanding, especially in the fields of population dynamics, reproduction and sexual selection, population and quantitative genetics, and behaviour, physiology and life history. We propose a number of future research opportunities using *Tribolium*, with particular focus on how their amenability to forward and reverse genetic manipulation may provide a valuable complement to other insect models.

## Introduction

Research models are fundamental to scientific investigation, providing simplified systems to test and explain components within more complex ideas and hypotheses (Hartmann and Frigg 2005). Model systems can be viewed along a continuum, where simplicity is traded against complex reality, with purely theoretical models at one end, and field-based systems at the other (Winther et al. 2015). Laboratory organisms occupy an intermediate position on this continuum, offering informative opportunities to directly test ecological or evolutionary theory in complex living systems, while retaining high levels of experimental control and allowing for experimental replication. As a result, laboratory models are an essential and widely used tool in biodiversity research, and here we discuss the many applications and strengths that *Tribolium* beetles possess for research in ecology and evolution.

**Fig. 1:**
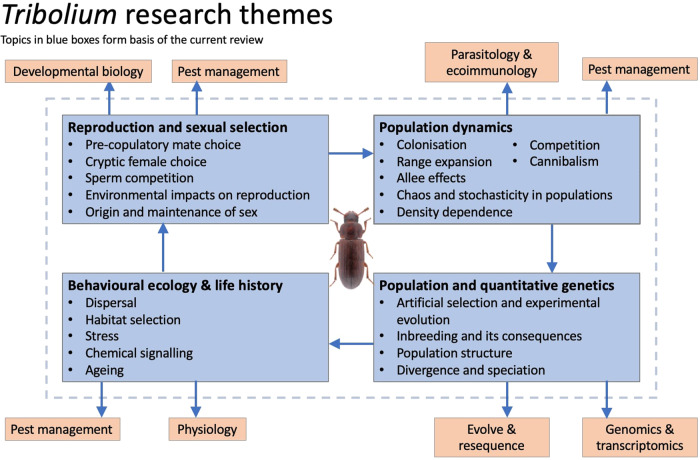
*Tribolium* research themes. Panels show interrelationships between research fields within evolution and ecology that have utilised *Tribolium* beetles as a research model. Those on blue backgrounds form the basis of this review (colour figure online).

In choosing experimental systems, scientists face a choice between focusing effort on one of rather few ‘true’ model organisms (Sommer 2009), versus adding diversity to the research base, expanding the useful extrapolations that can be made (Bolker 2012). Important distinctions exist between what some consider ‘true’ model organisms, compared to those used more broadly in experimental studies to which the term is often more loosely applied (Ankeny and Leonelli 2011). Under the more stringent ‘animal model’ view, the list of model organisms is traditionally limited to very few, including *Zea mays* (maize), *Arabidopsis thaliana* (thale cress), the bacterium *Escherischa coli*, *Saccharomyces cerevisiae* (yeast), the roundworm *Caenorhabditis elegans*, the fruitfly *Drosophila melanogaster*, *Xenopus laevis* (African clawed frog), *Mus musculus* (house mouse) and *Danio rerio* (Zebrafish) (Müller and Grossniklaus 2010). Attributes that distinguish these true model organisms include established research infrastructure, high experimental tractability and the ability to represent broad ranges of both taxa and questions under study (representational scope and representational target respectively, Ankeny and Leonelli 2011). Despite these nine classic models having such attributes, it is clear that we need to consider a wider range of models for progressing research, especially where we aim to tackle questions concerning the evolutionary ecology of biodiversity in the natural environment (Fig. 1).

**Fig. 2:**
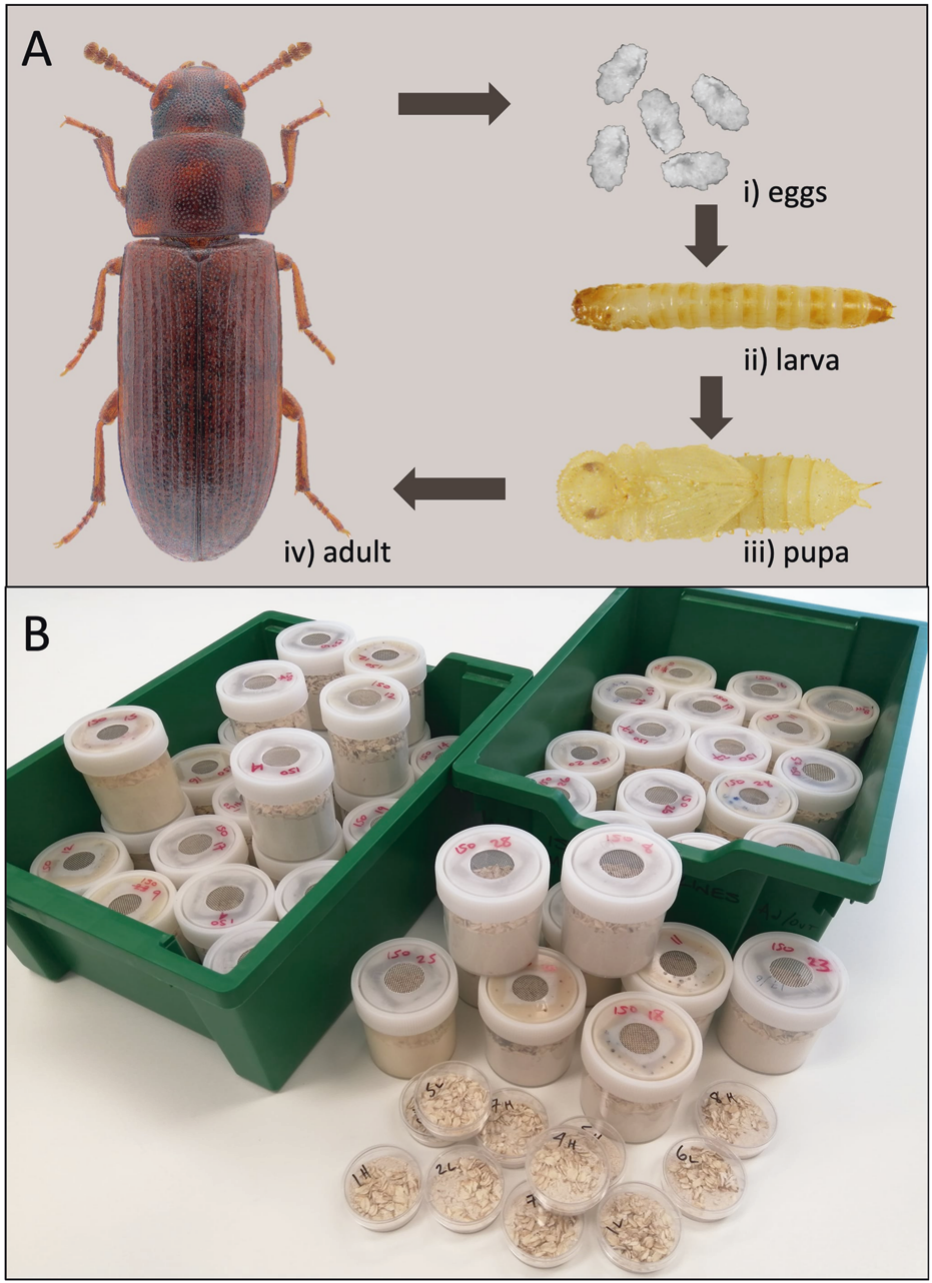
*Tribolium* life history and husbandry. **A** *Tribolium* *castaneum* life stages (compiled from photographs by Udo Schmidt (adult), John Obermeyer (egg), and from Khan et al. 2016). **B** Experimental *Tribolium* populations housed in 250 ml screw-top containers (top) and 50 mm Petri dishes, capable of supporting ~500 and ~100 individuals respectively without significant density-dependent effects.

*Tribolium* is a genus of small tenebrionid beetles, two of which, *T. castaneum and T. confusum*, are significant global pests of stored food products (El-Aziz 2011), and widely used as experimental models (Box 1; Fig. 2). Historically, *Tribolium* were free-living but are now mainly found infesting stored food products such as flour (Dawson 1977). The original habitat of *Tribolium* was likely beneath the bark of trees or in rotting wood, where they were secondary colonisers, characterised by rapid population growth and ready dispersal (Dawson 1977). It is not known when these pest species made the switch to a commensal lifestyle with humans, but there is evidence of *T. confusum* from Ancient Egyptian flour urns dating back ~5000 years, or ~70,000 beetle generations of selection (Andres 1931).

The purpose of this review is to highlight both the significant historical contribution from *Tribolium* flour beetles to ecological and evolutionary research, and elaborate their prominence as a contemporary study organism. *Tribolium* beetles have aided evolutionary ecology research for over a century (Chapman 1918), notably in population biology and interspecific competition (Park 1962), but also across a range of ecological and genetic disciplines. As members of the most species-rich order (Stork et al. 2015), occupying a relatively basal position among the Holometabola (metamorphosing insects), and being less highly derived than *Drosophila* (the foremost insect model), *Tribolium* beetles have broad representational scope (Brown et al. 2003). In addition, *Tribolium* is highly amenable to both forward and reverse genetic manipulation (Brown et al. 2009), a key attribute of any modern model organism (Barr 2003). *T. castaneum* was the first Coleopteran, and first agricultural pest, to have its genome sequenced (Tribolium Genome Sequencing Consortium et al. 2008), and annotations continue to be updated (Herndon et al. 2020). With the increasing availability of genomic data, biology is likely to become more comparative across models (Hedges 2002). *Tribolium* occupies a unique position to act as a mediary in the identification of insect orthologs of human genes, breaking a previous reliance on candidate genes from *Drosophila* and expanding the scope of insect genetics beyond highly conserved regions (Brown et al. 2003). While the *Tribolium* research infrastructure is certainly less well established than those of *Drosophila* and *C. elegans*, it is considerable and growing, with stock centres providing wild-type and many mutant strains to researchers (Brown et al. 2009), and increased availability of genomic and reverse genetic resources (Kim et al. 2010; Dönitz et al. 2015). Finally, this model is also a pest that significantly impacts global food supplies, and understanding gained in the laboratory can be applied to increase global food security (El-Aziz 2011).

Reviews of the *Tribolium* system exist, but most cover fields such as biomedicine, physiology and particularly developmental biology, where *Tribolium* is an important model due to its more representative mode of short germ band development than alternative insect models (Denell 2008; Adamski et al. 2019). Other fields where *Tribolium* are used, including parasitology, ecoimmunology, the evolutionary ecology of infectious disease and applied pest management, have relevance to the areas discussed, though we lacked the space to include them here (e.g., Kerstes and Martin 2014; Perkin and Oppert 2019; Eggert et al. 2015). Broad reviews of *Tribolium* as a model in ecology and evolution also exist but are several decades old (e.g., King and Dawson 1972; Mertz 1972; Sokoloff 1977) and much new research in this area has since emerged. We therefore aim to demonstrate that the *Tribolium* literature testifies to the model’s applicability across ecological and evolutionary fields and levels of biological organisation. We break the review down into conceptual areas, focusing on: (1) population dynamics, (2) reproduction and sexual selection, (3) population and quantitative genetics, and (4) behavioural ecology, ecophysiology and life-history evolution. We also discuss implications of *Tribolium* research findings for the broader ecological and evolutionary community, and suggest some ways in which this system can continue to enhance ecological and evolutionary research. We hope that this review will be valuable both to those within the *Tribolium* community, by providing context to the often highly specialised focus of individual research groups, and to a broader readership, by synthesising the significant contributions made to ecological and evolutionary science by this often-overlooked model species.

Box 1. *Tribolium* ecology and experimental tractability Many aspects of *Tribolium* ecology make them well suited as experimental organisms:

- The *Tribolium* environment is also the food medium: a dry mix of fine particulates that is easily prepared and readily stored. Homogeneity within this medium eliminates micro-climatic and nutritional variation within and between replicate populations, allowing tight environmental control of both temperature and humidity, the latter being important in reducing the invasion of fungal pathogens.
- Owing to *Tribolium’s* long human-commensal history (~70,000 generations), the laboratory medium also has the advantage of very closely approximating its semi-wild habitat in food-storage facilities, allowing a lab environment that is less abstracted than that in other insect models.
- Egg-to-adult development is completed in ~30 days (at 30 °C), with roughly 3, 20 and 4 days spent as egg, larva and pupa, respectively. Husbandry is therefore less tightly time constrained compared with faster-developing species, allowing more flexibility to generate high replicate numbers.
- Adults are small (~4 mm) but dark in colour and easily distinguished from the medium. All stages, including eggs can be separated from the medium by simple sieving for counting or other experimental manipulations.
- Individuals are physically and behaviourally robust to handling, without the need for anaesthetisation, allowing methods such as paint-marking of individuals for later identification.
- The sexes can be distinguished in adults by the presence of a gland on the forelimbs of males, which is absent in females. Pre-reproductive individuals are most easily sexed as pupae, by their differential genital morphology, allowing single-sex cohorts or virgins to be isolated for breeding and mating studies.
- Though able to fly, individuals display very limited motivation to do so in the laboratory, making containment simple relative to insects that fly. Under specific conditions, however, flight behaviour can be induced in *Tribolium*.
- *Tribolium* mate and breed readily and promiscuously in the lab as pairs or groups, enabling manipulation of mating pattern, high levels of success in specific breeding crosses, and good opportunities for the recording of reproductive behaviour.

## Population dynamics

The value of the *Tribolium* system in answering questions in population biology was recognised early in the twentieth century (Chapman 1918; Park 1934), and the system is well known for its contribution to this field. Here we break this research down into that covering (1) colonisation dynamics, (2) drivers of population size and regulation, and (3) competition. We focus on the empirical literature, but it is worth noting that *Tribolium* experiments have contributed to a large body of important mathematical modelling work aiming to predict population dynamics (Mertz 1972; reviewed in Costantino et al. 2005).

### Colonisation and spread

Colonisation events are necessarily rare and therefore difficult to observe in nature, despite their importance. Lab models give us the opportunity to study and replicate events that determine the genetic and phenotypic make-up of contemporary populations. *Tribolium* life history is characterised by repeated episodes of dispersal and colonisation (Dawson 1977), making it an ideal system in which to investigate these processes in the laboratory. For instance, while it is well understood that the size and frequency of colonisation events are positively correlated with colonisation success (Lockwood et al. 2005), the role of genetic and demographic processes in underpinning this relationship is less clear. *Tribolium* experiments have shown that both the frequency and size of introductions affect the likelihood of establishment, with some evidence that the former may be more important (Koontz et al. 2018). Further, by manipulating levels of genetic diversity within colonising populations, an elegant set of *Tribolium* experiments have shown that demographic and genetic processes have independent roles in determining colonisation success, by affecting initial establishment success and subsequent population growth respectively (Szűcs et al. 2014; Szűcs and Melbourne et al. 2017). Similar experiments have shown that the colonising individuals’ relative fitness in the new environment is as important as the number of colonisers (Vahsen et al. 2018), and that this affects population dynamics for many generations post-colonisation (Van Allen and Rudolf 2013).

Analysing the factors that underpin initial rates of population growth has also been a major aim of *Tribolium* research. It was in analysing *Tribolium* data that W.C. Allee formulated the theory that has become known as the ‘Allee effect’, an optimum density for initial population growth (Allee 1931; Park 1932), whereby at small population sizes mean individual fitness is reduced (Stephens et al. 1999). The rate of population growth has also been quantified across a range of environmental factors, including food quality and composition (e.g., Wong and Lee 2011), as well as life history parameters such as development time, with populations of fast-developing individuals able to grow more quickly than those in which individuals develop more slowly (Soliman 1972).

*Tribolium* is also well suited to the study of range expansion under a laboratory setting, using replicated populations within arrays of connected habitat patches. With such methods it has been shown that neutral stochastic processes are highly important in range expansion (Weiss-Lehman et al. 2019), with endogenous variation leading to highly unpredictable rates of spread (Melbourne and Hastings 2009). By experimentally constraining evolution, this work has shown that adaptation also plays a key role during expansion and the early stages of colonisation (e.g. Szűcs et al. 2017), though subsequent gene flow may be required into small populations to mitigate drift load which may hinder adaptation (Stewart et al. 2017). Addressing this same issue from a conservation perspective, experimental tests of both demographic and genetic rescue have shown that these interventions can reduce extinction risk and have additive effects (Hufbauer et al. 2015).

### Population size and regulation

A large body of experimental research has used *Tribolium* populations to determine the drivers of equilibrium population size and dynamics. Early *Tribolium* work showed that equilibrium population size is mediated by habitat volume (Chapman 1928). *Tribolium* researchers have since utilised the system to investigate questions regarding effective population size, including its response to population properties such as initial density (Wade 1980a) and its relationship to census population size (Pray et al. 1996).

Studies that have paired modelling approaches with *Tribolium* population biology experiments have added greatly to our understanding of population dynamic phenomena, directly linking theory to the behaviour of real populations (reviewed in Cushing et al. 2002; Costantino et al. 2005). Demographic parameters, predicted by models to lead to chaotic dynamics, were applied experimentally to *Tribolium* populations, exposing chaotic dynamics that could be reliably disrupted by minor intervention (Costantino et al. 1995, 1997; Desharnais et al. 2001). Comparing *Tribolium* population dynamic data to model predictions has further revealed influences of stochastic processes in the behaviour of populations, as well as identifying lattice effects (dynamics arising due to the discrete nature of individual organisms, e.g., Henson et al. 2001; King et al. 2004) and accounting for the ecological synchrony between separate populations (Desharnais et al. 2018).

*Tribolium* populations self-regulate via complex interactions of density-dependent effects on reproductive, developmental, dispersive and cannibalistic behaviours (King and Dawson 1972). Chemical secretions (see ‘life-history’ section, below) accumulate in the medium in proportion to time and density in a process known as ‘conditioning’, and these secretions are used by the beetles as indicators of demographic parameters, such as population density (El-Desouky et al. 2018). Much of the work on this area was done in the early-mid nineteenth century and is well covered by King and Dawson (1972). Oviposition is suppressed by conditioning of the medium and increased by egg cannibalism (Sonleitner and Gutherie 1991). Recent work has shown that females evaluate current and future competitive conditions when making oviposition decisions (Halliday et al. 2019). Larval development is slowed by increasing larval and adult density (Park et al. 1939; Janus 1989). Higher density during development also has indirect negative effects on future reproductive success through reduced body weight (Assie et al. 2008). Dispersal increases with density (Ziegler 1978) and there is evidence that the genetic bases of dispersal and the reduction of oviposition in response to conditioning are linked (Lavie and Ritte 1980, see also ‘Life history’ below). Density-dependent processes in *Tribolium* are complex and act differentially on the sexes (Ellen et al. 2016), interact with food quality (patterns of density-dependent habitat selection depend on the foodstuffs used, Halliday et al. 2019) and weaken with deviation from thermal optimum (Halliday and Blouin-Demers 2018).

Cannibalism is consistently shown to be core to many aspects of the *Tribolium* system, including population regulation. The degree of cannibalism increases with density until a point at which the predatory individuals become satiated (Park et al. 1965). Adult cannibalism of pupae is most effective at controlling adult numbers (Young 1970), while more nutritional benefit is gained from egg-eating (Alabi et al. 2008). Females are more voracious than males, and there is no reduction in cannibalism with increasingly conditioned medium (Flinn and Campbell 2012). The extra nutrition gained from cannibalism is able to compensate for the negative effects of high density (Mertz and Robertson 1970), and to facilitate colonisation of marginal nutritional habitat (Via 1999). Populations differ in their cannibalism rates, and these differences appear stable over many tens of generations; this may be because differences in cannibalism confer no selective advantage, or perhaps because populations can occupy different peaks on the selective landscape (Stevens 1989). Kin-selection may provide a mechanism for changes in cannibalism, as reduced rates of cannibalism between certain life stages have been observed within groups of highly related individuals (Wade 1980b).

Cannibalistic behaviour creates the periodic cycles in age-structure that characterise *Tribolium* populations (Costantino and Desharnais 1991) and, by manipulating population conditions, cycles can be altered or interrupted. When cannibalism is negated, by housing life stages separately, cycles disappear (Benoit 1998) and if populations are unconfined, allowing emigration, generations become non-overlapping (Ziegler 1972). These cycling phenomena are another area in which *Tribolium* has fostered close ties between theoretical and population biologists, by providing a convenient system in which to test theories of population dynamics, such as how environmental fluctuation affects population cycling (Reuman et al. 2008).

### Competition

Much of the reputation of the *Tribolium* system is founded on the two-species competition experiments of Thomas Park and his collaborators and their indeterminate outcomes, which helped draw attention to the role of stochastic processes in ecology (Park et al. 1964). Briefly, using experimental populations containing both *T. confusum* and *T. castaneum* it has been shown that (i) one species is almost always driven to extinction; (ii) the ‘winning species’ cannot be predicted by the relative performance of each species cultured alone in the focal environment; (iii) outcomes can be indeterminate under certain conditions, whereby the ‘winner’ is not the same in all replicates; (iv) there was no evidence for an elevated ‘win rate’ of populations which had won in previous experiments, suggesting that competitive ability cannot be selected on (Park and Lloyd 1955). These results spawned much work trying to account for indeterminate outcomes, including mathematical modelling (e.g., Leslie 1962). Following this early work, competition experiments in *Tribolium* have shown that demography may be more important than genetics in determining competitive outcomes in this system (Mertz et al. 1976), although inbreeding depression can cause a loss of competitive ability at low founding size (Craig and Mertz 1994). Further, a wide range of external variables have been shown to affect competition dynamics, including reduced competitive ability resulting from parasite infection and/or low natal habitat quality, and deviation from the thermal optimum (e.g., Yan et al. 1998; Van Allen and Rudolf 2015).

## Reproduction and sexual selection

One of the most fundamental life processes is reproduction, yet much is still unknown about the origin and maintenance of sex, and the evolutionary forces that maintain the diversity of reproductive phenotypes observed in nature (Williams 1975). To address these, and related questions has been a rich area of *Tribolium* research. Indeed, *T. castaneum* was used in one of the first ever sperm competition and fertilisation precedence experiments (Schlager 1960). Previous broad reviews have identified ways by which females may influence paternity during and following mating, including: inhibiting sperm transfer; altering re-mating behaviour; controlling timing of spermatophore ejection (Pai and Bernasconi 2008; Fedina and Lewis 2008). These reviews also cover the male traits shown to affect paternity share in the many studies of sperm precedence conducted on *Tribolium*. We will therefore focus on subsequent advances in these areas.

Experimental studies have provided varied insights into the evolution and ecology of mating behaviour. Recent work on pre-copulatory behaviour has shown that females exhibit a preference for non-stressed males (McNemee and Marshall 2018) and that homosexual behaviour, which is quite common in *T. castaneum*, is dependent on the social environment and likely occurs due to inaccurate mate choice (Martin et al. 2015; Sales et al. 2018). Post-copulatory reproductive processes are also an important area of research, and there is much potential to track the dynamics of sperm behaviour and male–male interactions within the female tract in vivo. Advances in fluorescent tagging of sperm have made it possible to visualise sperm fate following natural matings and their movement through the tract to the fertilisation set (Droge-Young et al. 2016), and molecular methods have facilitated the study of seminal fluid proteins (South et al. 2011).

Many aspects of reproduction appear to be environment dependent, with factors such as nutrition, temperature, conditioning of medium and parasite presence shown to alter mating dynamics and reproductive fitness, both individually and in combination (Grazer et al. 2014; Khan et al. 2018; Sales et al. 2018; Vasudeva et al. 2019). Further, reproductive fitness has been shown to trade off with intrinsic factors such as walking ability (Matsumura et al. 2019) but can be enhanced by others, such as pesticide resistance (Arnaud et al. 2005). Phenotypic plasticity and genotype-by environment interactions also play an important role in determining levels of sexual conflict and adaptability (Lewis et al. 2012; Holman and Jacomb 2017).

*Tribolium* experiments have been used to address fundamental questions about the ‘sex paradox’, sexual selection and the origin of mating behaviours (Dunbrack et al. 1995). Key to these studies has been the experimental manipulation of sex ratios and mating patterns over one or multiple generations, thereby applying different sexual selection pressures, and then measuring the consequences. Using such approaches, it has been shown that levels of promiscuity increase following genetic bottlenecks, and therefore that promiscuity may provide a mechanism for avoiding genetic incompatibility (Michalczyk et al. 2011). Using long-term sexual selection lines and experimental inbreeding, *Tribolium* experiments demonstrated that sexual selection may buffer populations against extinction through purging of mutation load (Lumley et al. 2015). However, subsequent studies using alternative approaches to examine the relationship between sexual selection and mutation load have found no purging effects (e.g. Prokop et al. 2019). A population history (up to ten years) of experimentally applied, strong sexual selection has also been shown to improve the competitive ability of males and their sperm, and drive sperm morphology evolution (Michalczyk et al. 2011; Demont et al. 2014; Godwin et al. 2017), increase conspecific population invasion success (Godwin et al. 2018), enable population resilience to the extinction vortex (Godwin et al. 2020), and increase the rate of pesticide resistance evolution (Jacomb et al. 2016).

## Population and quantitative genetics

Laboratory insect models have been instrumental in developing our understanding of population genetic theory, from the tracking of inversions in populations (Wright and Dobzhansky 1946) to the discovery of genetic markers (Lewontin and Hubby 1966). Though relatively understudied in comparison to *Drosophila*, many of the same features of *Tribolium* that made it originally attractive to those studying population dynamics, also make it suitable as a lens through which to view evolution in experimental populations from a genetic perspective. Since Park’s competition experiments first drew the attention of geneticists towards the *Tribolium* system, genetic investigations have formed an important branch of its study (King and Dawson 1972). Genetic work on *Tribolium* spans levels of organisation, covering everything from the relationship of genotype to phenotype, through to interspecific reproductive isolation.

### Artificial selection and experimental evolution

Early studies of adaptive evolution in *Tribolium* were based on observing phenotypic changes over generations, revealing that changes (in traits such as development time or pupal weight) can arise due to variation in productivity and cannibalism (Sokal and Sonleitner 1968; King and Dawson 1972). Similar, approaches have been used to show that the spread of a selfish genetic element through a population is proportional to the strength of its effect (Wade and Beeman 1994), and that pesticide-resistant genotypes can have increased fitness, even in the absence of pesticides (Haubruge and Arnaud 2001).

The majority of experimental studies of adaptive evolution have directly manipulated the strength of selection. Selection can be artificially applied, with those individuals who will seed the next generation being chosen by the researcher based on a given trait. Alternatively, experimental evolution can be used, where natural selection is allowed to act within populations subjected to a treatment. These techniques enable, amongst other things, the study of the relationship of genotype to phenotype. Provided that the strength of selection is known, trait heritability can be inferred from the response to selection. Alternatively, crosses between individuals of different phenotypes can reveal the genetic basis of traits. In these ways, it has been shown that traits such as pupal and adult weight are highly heritable in *Tribolium* (Wade et al. 1996). Some have moderate heritability, such as larval weight, development time and walking distance (Yamada 1974; Matsumura and Miyatake 2015). While some, like fecundity and death-feigning duration, respond only weakly to artificial selection (Orozco and Bell 1974; Miyatake et al. 2004).

Response to selection has been shown not only in terms of the focal trait, but also in associated traits. For example, weight metrics show correlated responses to selection across life stages (Yamada 1974) and populations selected for short death-feigning duration also have short legs and lower walking motivation (Matsumura and Miyatake 2019). Lines selected for low dispersal propensity have greater longevity, poorer flight ability, are better competitors, develop more quickly and have longer generation times than dispersers (e.g., Zirkle et al. 1988). Some of these associations between traits can limit the response to selection (Irwin and Carter 2014), and attempts to break the correlations of pairs of traits through artificial selection have failed (Bell and Burris 1973; Tigreros and Lewis 2011).

Wright’s shifting balance theory (Wright 1932) suggests that population structure may also influence response to selection, although there is limited evidence that this process plays an important role in nature (Coyne et al. 2000). *Tribolium* studies have applied artificial selection within sub-divide-and-merge population structures to test predictions of shifting balance theory (see Wade and Goodnight 1998). However, large panmictic populations have been found to respond better to selection for pupal weight than sub-divided, periodically mixing ones (Katz and Enfield 1977) and no difference in response was found using a similar design to select for offspring number (Schamber and Muir 2001). Further, it has been suggested that these experiments do not capture the complexity of the shifting balance process, and that doing so experimentally may not be possible (Coyne et al. 2000).

In addition to studying adaptation, *Tribolium* experiments have been used to understand the causes and consequences of stochastic processes such as mutation and drift in evolution (e.g., Rich et al. 1979). Inbreeding depression has been studied in detail in *Tribolium*, and inbreeding has been shown to have negative effects on a range of traits, including productivity and viability (López-Fanjul and Jódar 1977). Susceptibility to inbreeding depression can vary between populations and affect suites of traits within populations (Pray and Goodnight 1995; Pray 1997). Inbreeding has negative consequences for population growth and response to selection (McCauley and Wade 1981; Wade et al. 1996). However, the fitness consequences of inbreeding may only be realised in certain environments (subject to genotype-by-environment interactions arising under inbreeding), and may differ between sexes (Pray et al. 1994). The above studies generated inbreeding through enforced sib–sib matings, however more ‘realistic’ approaches have also been utilised. For example, by reducing population size but allowing free matings, bottleneck scenarios can be simulated, these have shown that subsequent stress following a bottleneck can reveal inbreeding severity, even after population size has recovered (Franklin and Siewerdt 2011).

### Divergence and speciation

Genetic differentiation has been quantified empirically between wild *Tribolium* populations (Drury et al. 2009; McCulloch et al. 2019) and between lab populations of varying geographic origin (Yamauchi et al. 2018). While results conflict between studies, it is clear that at least some genetic structure exists within *Tribolium* species. Although there has been limited research into the drivers of population divergence, there is a substantial body of experimental research examining its consequences. Female *T. castaneum* have been shown to increase egg-laying rates when mated to inter-population males, suggesting potential inbreeding avoidance (Attia and Tregenza 2004). On the other hand, when the sperm of males from different populations compete for fertilisations, ‘home’ males can be seen to have an advantage, indicative of partial reproductive isolation (Pai and Yan 2002). Other inter-population crosses can result in perpetually immature larvae (Drury et al. 2011), and show genetic incompatibilities in *T. castaneum* that provide support for the Haldane’s rule (Demuth and Wade 2007). Partial reproductive isolation through mate choice has also been observed between populations of *T. confusum* (Wade et al. 1995).

Moving beyond populations, species across the *Tribolium* genus display a range of divergence times, making them useful for studies of reproductive isolation at the species level. Between *T. castaneum* and *T. confusum*, reproductive isolation is incomplete (Shen et al. 2016), while between *T. castaneum* and *T. freemani*, hybrids are sterile (Wade and Johnson 1994). Interspecific crosses have therefore been used to study questions related to speciation, showing for instance that significant post-copulatory prezygotic isolation can arise through asymmetric sperm precedence under interspecific male competition (Robinson et al. 1994). Crosses between *T. castaneum* and *T. freemani* have also shown that the degree to which skewed sex ratios and male deformity manifest in the F1 generation varies with the geographic origin of the *T. castaneum* strain used, even at relatively local scales (Wade et al. 1997).

The wide range of geographic and genetic distances between the species of *Tribolium*, means that the group constitutes an excellent system for comparative genomic studies seeking to understand the drivers and consequences of speciation (Brown et al. 2009), this will likely be a rich area of future study. Another area for expansion in *Tribolium* research is population genomics: the attributes that have seen the system used in population studies in the past, along with its emerging ability to generate high quality genomic data, are suited to combine in the study of genomic responses to selection. This could take the form of ‘evolve and resequence’ approaches that have so far struggled to make it beyond *Drosophila (*Schlötterer et al. 2015).

## Behavioural ecology and life history

Research on *Tribolium* began with studies of populations, but soon branched out to include the individual-level life-history traits that underlie the dynamics of populations, often in order to correctly parameterise mathematical models. Today *Tribolium* is a model for studying life-history parameters of invertebrates in its own right. Ongoing life-history studies in *Tribolium* can mostly be divided into those concerned with dispersal, movement and habitat selection; responses to environmental stress and diet; and studies of chemical biology and ageing.

### Dispersal, movement and habitat selection

The study of why individuals disperse, what biological and environmental factors drive this, and the population-level consequences of this process are important areas in evolutionary ecology (Bowler and Benton 2005). Laboratory insect models offer a useful opportunity to study dispersal, as individuals can be tracked, the process of dispersal can be measured in a controlled and repeatable way, phenotypes associated with dispersal can be artificially selected, and the consequences of dispersal measured. *Tribolium* offers a particularly useful model in this respect, as it has two modes of dispersal (walking and flying), and is characterised by a life history that likely requires dispersal between patchy habitats (Dawson 1977). As a result, *Tribolium* has been widely used to study a broad range of questions about the evolutionary drivers and ecological consequences of dispersal.

Like many areas of *Tribolium* research, work on dispersal began in the mid twentieth century, but has seen a modern renaissance. *Tribolium* flight is rare, and early work focused on movement by walking, defining dispersal as the tendency of adults to leave a patch of habitat within an experimental apparatus composing two connected habitat chambers (Prus 1963; reviewed in King and Dawson 1972). Variations on this set-up over many years have shown dispersal to be dependent on a complex of factors, principally an interaction between density (Zromska-Rudzka 1966) and age (see Ziegler 1976; Gurdasani et al. 2018), altered by population-age and relatedness structure (Ziegler 1978; Jasieński et al. 1988), food availability (Ziegler 1977), as well as the natal environment of a focal individual or a threshold proportion of its neighbours (Van Allen and Bhavsar 2014; Endriss et al. 2019). Adult dispersal tendency appears to have a strong genetic basis (Ritte and Agur 1977), but this tendency is not conserved within individuals between pre- and post-metamorphosis (Arnold et al. 2016). Tendency to disperse is more highly correlated with leg length than metabolic rate, indicating that dispersal phenotypes depend more on morphological than physiological traits in adults (Arnold et al. 2017). Dispersal rate and sensitivity of dispersal responses to age and environment are greater in *T. castaneum* than *T. confusum*, consistent with the former being a primary coloniser, following a more r-type strategy than that adopted by the latter (Ziegler 1976).

The last decade has seen significant attention paid to the study of flight behaviour of *T. castaneum*, with the influence of biotic and abiotic factors on flight responses being investigated in laboratory, wild and wild-caught populations. Relative humidity does not appear important, while wind speed and direction, temperature, light, resource provision, and quality, age and mating status have all been seen to alter flight initiation and/or duration (e.g., Drury et al. 2016; Gurdasani et al. 2019). Flight patterns appear to be crepuscular and show seasonality (Daglish et al. 2017; Rafter et al. 2019), though this pattern varies with latitude (Rajan et al. 2018). Consensus on the effect of sex on flight remains elusive, as the results of these studies do not agree on whether flight behaviour differs between males and females.

Movement in relation to habitat selection by different life stages has also been studied in *Tribolium*, which can occur either by walking on the surface of the medium, or tunnelling through it (Hagstrum and Smittle 1980). Larvae seeking pupation sites tend to move deeper into the medium, seeking warmth and low population density, even when this means using poor quality habitat (King and Dawson 1972; Mayes and Englert 1984; Janus 1989). Adults move in response to biotic factors, avoiding high density areas and highly conditioned media, and alter their behaviour according to their reproductive status (Naylor 1965; Wexler et al. 2017). Adult movement behaviour can also vary in response to abiotic factors, including temperature, humidity and habitat structure (Campbell and Hagstrum 2002; e.g., Halliday and Blouin-Demers 2017). Studies investigating the distribution of adults within a fodder mass have shown that fine-scale spatial and temporal structure exists, and that individual variation in patch exploitation can serve to maximise individual fitness (Surtees 1963; Campbell and Runnion 2003). This variation in movement behaviour in response to resource availability may differ when flying as opposed to walking, and between wild and laboratory populations (Ahmad et al. 2013; Ahmad et al. 2013).

### Other life-history features

*Tribolium* represents a good model for studying the effects of stress due to the ease of replicability, and as a model that has relatively few ethical issues surrounding its use. Stress, in the form of starvation, heat or cold shock, or combinations thereof (Shostak et al. 2015), has been experimentally applied to show negative effects on reproductive output and behaviour, movement patterns, and immune response (Sbilordo and Gräzer 2011; Eggert et al. 2015; Wexler and Scharf 2017). Alternatively, effects of tolerance to stress can be the response variable, and this has been used to show that the ability to tolerate stress is affected by a range of factors including parental age (Halle et al. 2015), thermal acclimation regime (Izadi et al. 2019) and rearing conditions (Scharf et al. 2015). Later-life effects of natal/juvenile stress have also been shown in *Tribolium*, with the natal environment affecting adult dispersal (Van Allen and Bhavsar 2014), competitive dynamics (Van Allen and Rudolf 2015), productivity and rates of cannibalism (Boyer 1976).

*Tribolium* excrete a range of chemicals into their environment, some of which are aggregation pheromones (mainly 4,8 dimethyldecanal, Suzuki 1980), whereas others are a defence against predators and microorganisms whilst playing an important role in population dynamics as indicators of population density (Arnaud et al. 2000). The chemicals used as pheromones differ across the genus (Arnaud et al. 2002), and are produced predominantly by males, eliciting the strongest responses from females (Stevenson et al. 2017). Diet alters the chemical composition of secretions, but may not alter their efficacy (Ming and Lewis 2010). Defensive compounds such as benzoquinones have antimicrobial properties (Prendeville and Stevens 2002) and show genetically controlled differential production across individuals (Yezerski et al. 2004). Due to the shared benefit of their action through density regulation, and the individual costs associated with their production, this can represent a social dilemma (Gokhale et al. 2017). The distinction between these two classes of secretions is not clear, with benzoquinones also shown to possess pheromone-like attractive properties (Verheggen et al. 2007). Understanding *Tribolium* chemobiology represents a fascinating future challenge, given the potential of secretions to influence individual, population and interspecies processes.

Considering its role as a pest, there has been much interest in the ability of *Tribolium* to utilise different food resources and the effect that these have on its life history. Early authors list a wide range of products on which *Tribolium* has been found, including flours of many grains, peas, beans, nuts, chocolate and several spices (e.g., Chittenden 1896). Grain preference was among the first tests performed by Chapman, the man credited with pioneering the use of *Tribolium* for experimental study, who found a preference for wheat flour, and an inability to feed on whole grains (Chapman 1918; Park 1934). Very many studies of responses to diet and diet quality in *Tribolium* have since been published, which show that natural or artificial diets result in large effects on a broad range of fitness parameters (e.g., Sinha 1966; Sokoloff et al. 1966; Wong and Lee 2011).

Finally, there is a limited body of research on ageing in *Tribolium. T. confusum* was the organism in which exposure to radiation was first shown to increase longevity (Davey 1917); this work was subsequently expanded on and replicated widely among insects (Ducoff 1986; Calabrese 2013). However, recent decades have seen *Tribolium* superseded as an invertebrate model of ageing by relatively short-lived alternatives in *Drosophila*, *C. elegans* and yeast (Kennedy 2008). Nonetheless, age-related changes have been shown in individual-level morphological, physiological, biochemical, behavioural and pathological traits (Soliman 1987). Ageing research in *Tribolium* has generated important insights into the link between development and ageing and their genetic basis and the evolution of senescence (Soliman and Lints 1975; Mertz 1975). Some more recent work has highlighted parental-age effects on development and stress tolerance (Halle et al. 2015) and a decline in metrics of movement with increasing age (Wexler et al. 2016). As a relatively long-lived arthropod model (adults can live for up to 4 years, Good 1936) *Tribolium* may have utility for understanding ageing in slower-senescing animals such as vertebrates.

## Future directions and conclusions

We have highlighted some of the broad range of fields in which *Tribolium* has been successfully used as a research model. However, we believe that there is still much untapped potential from this organism for addressing several research areas, particularly the expansion of historically productive *Tribolium* fields in combination with genomic data. Studies of divergence can continue to exploit the genus’ diversity and mating ecology, in combination with genomic techniques, to probe deeper into the process of species formation. Population dynamic and artificial selection responses can be investigated at the level of the entire genome, expanding the complexity of our understanding. Pest management can employ genomic information to increase the efficacy and specificity of its techniques, minimising the collateral damage while maximising benefits.

Importantly, *Tribolium* research can also incorporate work on climate change, which represents an enormous threat to biodiversity, with insects likely to be severely affected due to their short life cycles and temperature sensitivity (Bale et al. 2002). There is great scope for *Tribolium* as a model in which to study the effects of climate change on insects, for example by studying their physiological, ecological and evolutionary responses to experimental evolution under increased temperature. Many of the characteristics lending research utility to *Tribolium* (Box 1) also make it highly suitable as a research-led teaching resource (Hoste 1968), a role in which we feel it has been underutilised in the past.

*Tribolium* beetles possess many attributes that have made them a desirable study organism through a long history and breadth of application. They have contributed to many important past discoveries, and continue to be employed in addressing fundamental questions across fields in evolution and ecology. The utility of *Tribolium* spans levels of organisation, and a great responsiveness to genetic manipulation (Brown et al. 2009) promises to extend their relevance far into the genomic age. The ever-growing research infrastructure, and ability to integrate knowledge from across fields, makes *Tribolium* a valuable model system to complement the established invertebrate models of *Drosophila* and *C. elegans*.
